# Role of Signaling Transduction Pathways in Development of Castration-Resistant Prostate Cancer

**DOI:** 10.1155/2011/647987

**Published:** 2011-10-05

**Authors:** Takahiro Inoue, Osamu Ogawa

**Affiliations:** Department of Urology, Graduate School of Medicine, Kyoto University, 54 Kawaharacho Shogoin, Sakyo-ku, Kyoto 606-8507, Japan

## Abstract

Almost all patients who succumb to prostate cancer die of metastatic castration-resistant disease. Although docetaxel is the standard treatment for this disease and is associated with modest prolongation of survival, there is an urgent need for novel treatments for castration-resistant prostate cancer (CRPC). Great advances in our understanding of the biological and molecular mechanisms of prostate cancer progression have resulted in many clinical trials of numerous targeted therapies. In this paper, we review mechanisms of CRPC development, with particular focus on recent advances in the understanding of specific intracellular signaling pathways participating in the proliferation of CRPC cells.

## 1. Introduction

Prostate cancer is the most common malignancy and the second leading cause of cancer death in the United States [1]. The American Cancer Society has estimated that 217,730 new cases will be diagnosed and 32,050 deaths will occur in the United States in 2010. However, in Japan, the incidence of prostate cancer was still lower than that of gastric and lung cancer in 2005 but has been markedly increasing, and it is predicted that the incidence will be second to lung cancer by 2020 [2]. In the early 1940s, Huggins demonstrated that the growth and survival of prostate cancer are dependent upon androgens [3]. Most patients with advanced prostate cancer initially respond to androgen ablation therapy. However, after an initial period of response to this therapy most of these patients finally relapse and develop castration-resistant prostate cancer (CRPC). 

 CRPC cells mostly continue to express androgen-regulated genes, such as PSA, despite greatly reduced levels of androgens. In particular, androgen receptor (AR) expression is required for survival, and the AR signal axis is still activated in this state. Several possible mechanisms have been proposed, including: (a) the development of hypersensitive response due to increased AR levels or *de novo* production of endogenous androgens within the prostate; (b) promiscuous activation of AR due to mutation in ligand binding domain of AR; (c) altered transcriptional activity of AR due to changes in expression of coactivators and/or corepressors; (d) ligand-independent activation of AR through growth signaling activation [4, 5]. Alternatively, other models suggest that preexisting androgen-independent cancer cells infrequently occur within tumors and undergo clonal selection during androgen deprivation therapy, resulting in CRPC [4]. 

## 2. PTEN-PI3K Signaling Pathway

In 1997, the tumor suppressor gene *PTEN* was identified as a gene that is mutated in multiple sporadic tumor types, as well as in patients with predisposed cancer syndromes, such as Cowden disease [6–8]. PTEN negatively regulates flux through the phosphatidylinositol 3-kinase (PI3K) signaling pathway [9], which plays a critical role in tumorigenesis in a variety of malignancies. Therefore, the PTEN-PI3K signaling pathway functions as a crucial regulator of cell survival fate. Mouse models have shown that *Pten* heterozygotes develop prostatic intraepithelial neoplasia (PIN), but these lesions do not progress to invasive cancers [10]. On the other hand, *Pten* heterozygosity cooperates with loss of other genes, such as *Nkx3.1* and *Cdkn 1b* (*p27*), in the development and progression of prostate cancer [10]. Conditional knockout mice, lacking both alleles of *Pten*, develop invasive prostate cancer [10]. In human prostate cancer, loss of the *PTEN* gene occurs in a substantial proportion of metastatic cancer and in approximately 20% of locally advanced lesions [11]. By contrast, only a minority of low-grade and low-stage tumors harbor *PTEN *mutations [12]. Therefore, these data strongly suggest that PTEN has a role as a tumor suppressor in prostate cancer initiation and progression. 

Use of a prostate-specific *Pten *deletion mouse model and PTEN knockdown in cell lines revealed that loss of PTEN promotes resistance to castration [13]. In spite of this evidence, there are few studies that clearly explain the mechanism. Interestingly, Carver et al. recently showed that the PI3K pathway and AR signaling regulate each other by reciprocal negative feedback, so that inhibition of one activates the other [14]. They showed by mRNA transcriptome analyses that *PTEN* loss strongly correlated with repression of AR activity. Moreover, they revealed that inhibition of the PI3K signaling pathway restores AR signaling in PTEN-deficient prostate cancer cells, suggesting that resistance to castration can be reversed. One mechanism of AR signaling activation by PI3K inhibition seems to be through relief of negative feedback inhibition to HER kinases. Additionally, blockade of AR pathways attenuates FKBP5 expression, which is androgen dependent in prostate cancer cells, resulting in increased AKT phosphorylation due to a reduction in PHLPP protein levels since FKBP5 is a molecular chaperone for the AKT phosphatase PHLPP. In their study they used BEZ235, a dual inhibitor of PI3K and mTORC1/2. Thus, BEZ 235 treatment in PTEN-deficient prostate cancer cells does not delineate which target is more potent for AR signal activation. This reciprocal negative feedback regulation between androgenic and PTEN loss/PI3K-AKT signaling in prostate cancer has also been reported by other groups [15]. However, according to previous experiments, activation of the mTOR signaling pathway in LNCaP cells represses PSA expression [16]. Conversely, treatment with rapamycin, an mTOR inhibitor, increases PSA expression [16]. Since PI3K regulates mTOR signaling activity, this evidence shows that the PI3K/mTOR pathway negatively regulates AR signal activity. Therefore, this bidirectional crosstalk between these two critical survival pathways in prostate cancer provides a reasonable rationale for simultaneously inhibiting both signaling pathways [17, 18]. Additionally, evaluation of PTEN status and appropriate AR pathway inhibition, together with treatment with PI3K/mTOR inhibitor, could shed a ray of hope in overcoming treatment of CRPC. 

## 3. Protein Kinase C and Prostate Cancer

Protein kinase C (PKC) isoforms are serine/threonine kinases involved in the transduction of a significant number of signals for the regulation of cell proliferation, differentiation, tumorigenesis, apoptosis, and cytoskeleton remodeling [19, 20]. The PKC isozymes comprise a large family, which are grouped by three subfamilies subdivided into conventional, novel, and atypical isoforms according to their structure and mechanisms of regulation. The conventional PKCs, including PKC*α*, PKC*β*I, PKC*β*II, and PKC*γ*, are activated by calcium, phosphatidylserine (PS), and diacylglycerol (DAG). The novel PKCs, including PKC*δ*, PKC*ε*, PKC*η*, and PKC*θ*, require only PS and DAG for their activation. The atypical PKCs, such as PKC*λ*/*ι* and PKC*ζ*, require only PS to activate them [19–21]. 

The fact that PKC is a cellular receptor for the tumor-promoting phorbol esters led us to consider and investigate the role of individual PKC isozymes in carcinogenesis [22]. Metzger et al. recently reported that PKC*β*I phosphorylates histone H3 at threonine 6 (H3T6) and inhibits lysine-specific demethylase 1 (LSD1), which is a component of corepressor complexes of gene transcription, from demethylating H3K4 during AR-dependent gene activation [23]. They showed that levels of PKC*β*I and phosphorylated H3T6 expression positively correlate with high Gleason scores of prostate cancer. Furthermore, inhibition of PKC*β*I attenuates prostate cancer proliferation both* in vitro* and *in vivo*. 

Hafeez et al. revealed that deletion of PKC*ε* in TRAMP mice inhibits prostate cancer development and metastasis through downregulation of prostatic STAT3 activation and STAT3-regulated gene expression [24]. A transgenic mouse model in which PKC*ε* was expressed specifically in the mouse prostate leads to the occurrence of prostate intraepithelial neoplasia (PIN) through STAT3 activation, suggesting that PKC*ε* may be involved in prostate cancer development [25]. 

## 4. Atypical PKC and CRPC

The atypical protein kinase C (aPKC) subfamily is composed of two members, PKC*λ*/*ι* and PKC*ζ*. PKC*λ* is the mouse homolog of the human PKC*ι*. The two isoforms are highly related and show 72% amino acid similarity [26]. There is growing evidence that aPKCs have been implicated in many biological processes, including cell survival and proliferation, cell polarity, and migration [5, 26]. Recently, several articles showed participation of aPKCs in prostate cancer development and progression. Par-4 binds both aPKCs and inhibits the enzymatic activity [5, 26]. The* Par-4* gene was originally identified in prostate cells undergoing apoptosis following androgen deprivation [5, 26]. *Par-4* KO male mice showed a high incidence of prostate hyperplasia and PIN [27]. *Par-4* KO male mice were sensitive to testosterone-induced prostate hyperplasia [27], and this hyperproliferation and development of prostate neoplasia can be blocked by concomitant attenuation of PKC*ζ* in mice [28]. In human prostate cancer specimens, a significant and direct correlation between Par-4 and PTEN protein levels was observed. Most Par-4 positive tumors were also positive for PTEN, whereas those tumors with negative or low Par-4 expression also exhibited negative or low expression of PTEN. PTEN inactivation has been found to be associated with aggressive and high Gleason scoring cancers. Similarly, there was a correlation between Par-4 loss and high Gleason scores. Interestingly, concomitant loss of Par-4 in the context of PTEN heterozygosity resulted in invasive prostate carcinoma in mice through activation of Akt and a synergistic stimulation of NF*κ*-B [28]. These observations suggest that Par-4 and PTEN have a strong cooperative effect on human prostate carcinogenesis. 

All these findings suggest that aPKCs may have roles in carcinogenesis and progression of prostate cancer. 

We have established AILNCaP cells, which are derived from LNCaP cells after long-term culturing under androgen-deprived conditions. They can proliferate without androgens, and thus this transition model mimics the clinical status of patients who eventually become resistant to castration. Using this model, we identified that activity of S6K signaling pathway is regulated by androgen/AR signal, and activation of the S6K signaling pathway is required for proliferation of LNCaP cells [29]. Moreover, its constitutive activation is also required for proliferation of AILNCaP cells under androgen-deprived conditions. Through investigation of various signaling pathways participating in activation of S6K pathway, we clarified that aPKCs activity regulates the upstream signal of this pathway [29]. In LNCaP cells, androgen deprivation reduced the amount of activated form of aPKCs. In contrast, activated forms of aPKCs could be seen in AILNCaP cells without androgen stimulation. Moreover, androgen stimulation upregulated the degree of activated forms of aPKCs in LNCaP cells. We also found that exogenously introduced wild-type aPKCs were associated with S6K in prostate cancer cell lines and that this association correlated with androgen stimulation. Inhibition of aPKCs using a specific inhibitory peptide attenuates the activated form of S6K both in LNCaP cells and AILNCaP cells. The same trend was observed in the activated form of the downstream target S6, and the expression of cyclin D1 was reduced in both cell lines. Cell cycle and morphological analysis revealed that inhibition of this pathway not only reduces cell proliferation but also induces apoptosis. The same observation can be seen in the AR-negative prostate cancer cell lines, PC3 and DU145. Thus, the aPKC/S6K signaling pathway participates in androgen-dependent proliferation of LNCaP cells, and constitutive activation of this pathway is required for cell proliferation and survival of androgen-independent prostate cancer cells [29]. 

**Figure 1:**
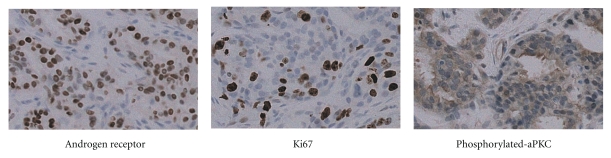
Representative immunohistochemical staining of castration-resistant prostate cancer tissues by specific antibodies against androgen receptor, Ki67, and phosphorylated atypical PKC.

**Figure 2:**
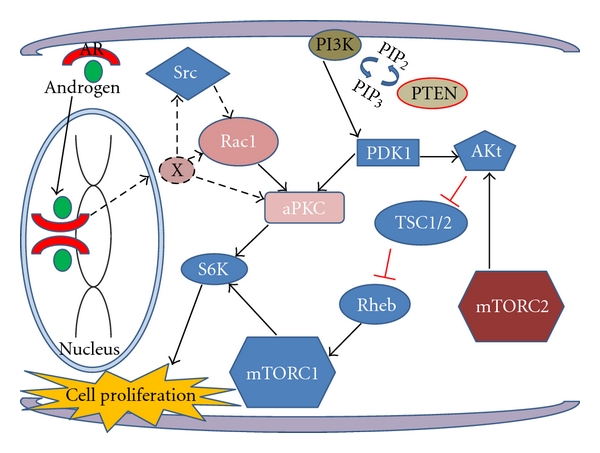
Intracellular signaling pathways including Rac1/atypical PKC (aPKC)/S6K pathways and androgen signaling. An unknown molecule (X) downstream of androgen/androgen receptor (AR) may activate Rac1 and Src (depicted as dotted arrows) and may contribute to androgen-dependent cell proliferation. In castration-resistant prostate cancers, AR activity is upregulated by specific mutations for a promiscuous ligand-dependent manner or by other mechanisms, resulting in constitutive activation of Rac1/aPKC/S6K signaling through the same “X” or others.

Investigation of upstream regulators of aPKCs/S6K pathway revealed that Rac1, a member of Rho family small guanosine triphosphatases (GTPases), is a critical regulator for activation of the pathway [30]. Androgen stimulation increased expression of Rac1-GTP, which is an activated form of Rac1, and in contrast, androgen withdrawal attenuated the amount of Rac1-GTP form in LNCaP cells. Moreover, in this experiment, the amount of Rac1-GTP correlated with the activated forms of aPKCs and S6K, and inhibition of Rac1 with pharmacological and genetic methods induced attenuation of the aPKCs/S6K signaling pathway. Furthermore, treatment with Rac1 inhibitor significantly inhibited the effect of androgen on cell cycle progression of LNCaP cells [30]. Collectively, these results suggest that the Rac1-aPKC-S6K signaling pathway is activated by androgen stimulation, and activity of this pathway is required for cell cycle progression of LNCaP cells. Introduction of constitutively active mutant Rac1 (Rac1V12) in LNCaP cells induced proliferation under androgen-depleted conditions both *in vitro *and *in vivo* [30]. Evaluation of Rac1 activation of syngeneic androgen-independent sublines of LNCaP cells, C4-2, and AILNCaP cells, under androgen-depleted conditions, revealed that Rac1-GTP is more abundant in these cell lines compared with that expressed in LNCaP cells cultured in the same conditions. Accordingly, both cell lines expressed increased levels of the activated forms of aPKCs compared with the parental cells. Attenuation of Rac1-GTP expression by pharmacological and genetic methods in C4-2 and AILNCaP cells reduced cell cycle progression and induced apoptosis, indicating that activity of Rac1 is related to androgen-independent progression and survival of LNCaP cells. Moreover, PC3 and DU145 cells expressed more Rac1-GTP compared with LNCaP cells, and attenuation of Rac1-GTP expression by pharmacological and genetic methods reduced cell cycle progression and induced apoptosis in both cell lines [30]. In human prostate cancer tissues, Rac1/aPKCs/S6K signaling is more highly activated in CRPC specimens in comparison to hormone-naïve cancers, as shown by immunohistochemical analysis (Figure 1). Collectively, these results suggest that the Rac1/aPKCs/S6K signaling pathway is required for progression to androgen-independent states, and thus this signaling pathway may be a potent therapeutic target of CRPC [30] (Figure 2). 

Interestingly, Ishiguro et al. have reported that PKC*λ*/*ι* expression correlates with biochemical failure after radical prostatectomy [31]. Additionally, they found that suppression of PKC*λ*/*ι* in DU145 cells attenuates their growth *in vitro* and *in vivo* and showed evidence that PKC*λ*/*ι* mediates IL-6 gene transcription through NF*κ*B and AP1 in prostate cancer cells. They finally concluded that overexpression of PKC*λ*/*ι* may participate in the progression to CRPC. Yao et al. have demonstrated that expression of PKC*ζ* in human prostate cancer specimens highly correlated with existing prognostic markers, such as Gleason score and clinical stage [32]. They also reported that suppression of PKC*ζ* by siRNA in PC3-M cells reduced proliferation and invasive capacities. 

These observations implicate that aPKCs have important role in the occurrence and progression of prostate cancer and that this signaling pathway might be a target for CRPC.

## 5. Prostaglandin and CRPC

Inflammation has been considered to be a key player in prostate carcinogenesis, and dietary consumption of nonsteroidal anti-inflammatory drugs may reduce the risk of prostate cancer [33, 34]. In prostate cancer patients, a strong association between the levels of serum C-reactive protein (CRP) and serum PSA has been reported [35]. Recently, elevated plasma CRP level has been considered as a strong predictor of poor prognosis in patients with metastatic CRPC [35]. 

Prostaglandins (PGs) have been considered to play a role in the development and progression of many malignancies [36, 37]. PGs promote carcinogenesis by multiple mechanisms, such as enhancement of cell proliferation, inhibition of apoptosis, and promoting angiogenesis. COX, an endoperoxidase synthase, is the rate-limiting enzyme that catalyzes the conversion of arachidonic acid to PGs and related eicosanoids [35, 37]. COX consists of two isoforms: COX-1, which is constitutively expressed in variety of tissues and cell types, and COX-2, which is induced its expression by a variety of stimuli [35, 37]. Genetic and clinical studies suggested that increases in *COX-2* expression is a key factor in prostate carcinogenesis and COX2 inhibitors have been tested in the treatment and prevention of prostate cancer [35]. However, these approaches have met with limited success and sometimes with severe cardiovascular toxicities [38]. Prostaglandin E (PGE) and prostaglandin F (PGF) are the major PGs stimulating the proliferation of prostate cancer cells, and they act by binding to prostanoid receptors and G-protein-coupled membrane receptors [35]. PGE acts through four different receptor subtypes, EP1 to EP4 [39]. The intracellular signaling differs among these receptors; EP1 is coupled to calcium mobilization, EP3 inhibits adenylate cyclase, and EP2 and EP4 stimulate adenylate cyclase in various cell types [39]. Previous experimental studies have implicated that increased EP2 and EP4 expression is important in colorectal and prostate cancer progression [40, 41]. 

We have established and reported two novel prostate cancer xenograft models, KUCaP-1 and KUCaP-2 [42, 43]. KUCaP-2 tumors are derived from locally recurrent CRPC specimens, harbor wild-type AR, and express PSA [43]. They regress soon after castration and restore their ability to proliferate after 1 to 2 months without AR mutations, mimicking clinical transition to CRPC. Precise investigation of this model revealed that EP4 expression significantly increased with the development of castration resistance [43]. In human prostate cancer specimens, the EP4 expression level was significantly higher in CRPC rather than hormone-naïve prostate cancer, which is compatible with the expression in KUCaP-2 model. EP4 overexpression induced castration-resistant proliferation in LNCaP cells both *in vitro *and *in vivo* through activation of the EP4-cAMP-PKA-AR signaling pathway. Treatment with specific EP4 antagonist, ONO-AE3-208, in EP4-overexpressing LNCaP cells and KUCaP-2 cells resulted in attenuation of their proliferation under androgen-deprived conditions *in vivo*, suggesting that EP4 might be a novel molecular target of CRPC [43]. 

## 6. Intrinsic Activation of Androgen Receptor

Recently, multiple kinds of AR alternative splicing variants lacking various portions of the ligand-binding domain (LBD) were identified in human prostate cancer specimens [44, 45]. These ARΔLBD variants are considered to be constitutively active, so that AR signaling can be stimulated in the complete absence of ligand binding. The clinical relevance of these truncated ARΔLBD variants has also been reported. The elevated expression of AR splice variants showed a significant association with more rapid disease recurrence after radical prostatectomy, in comparison to patients with lower expression levels of the variant [44]. Evaluation of specimens derived from CRPC metastases revealed that variant mRNAs of any form were increased in these specimens in comparison to normal prostate tissue samples [46]. Interestingly, a recent report suggested that some AR variants promote castration-resistant growth by acting through full-length AR and not independently [47]. Therefore, this growth is inhibited by ligand binding domain-targeted antiandrogens, such as MDV3100, or by specific siRNA for full-length AR. Moreover, some AR variants displayed ligand-independent biological activity, such as AR-V7, whereas AR-V1 had no effect for castration-resistant tumor growth. Thus, the role of AR variants in CRPC is complex, and further investigation will be needed.

## 7. Crosstalk with AR Signaling and Other Transduction Pathways

Numerous papers have described crosstalk of AR signaling with other transduction pathways [4, 48]. Since there are over 500 putative protein kinase genes in the human genome, the complexity and variety of signals that can be transduced are enormous. 

Mutations of Src kinase have not been reported in human prostate cancer, but Src kinase is overexpressed and activated in metastatic or castration-resistant prostate cancer [49, 50]. The activation of Src kinase contributes to prostate carcinogenesis through activation of various signaling pathways, including RAS, PI3K-Akt, integrin-FAK, MAPK, and STAT3 signaling [51]. Several studies provide evidence that Src kinase can interact with AR signaling pathways. Guo et al. found that the Src kinase activity was elevated in all three hormone-refractory prostate tumor xenografts [50]. Additionally, Src-induced tyrosine phosphorylation of AR, especially AR Y534, was critical for AR activation, and Src-ARY534 phosphorylation was important for cell proliferation under androgen-depleted conditions in prostate cancer cells. We reported that Src kinase activity was regulated by androgen stimulation through AR in LNCaP cells [30]. Moreover, Cai et al. recently suggested that wild-type Src kinase and AR mutually coactivate one another, and this interaction contributed to prostate cancer progression *in vivo* models [52]. Therefore, inhibition of Src kinase is now considered as a promising therapeutic target of prostate cancer, and dasatinib, the dual Src and Bcr-Abl tyrosine kinase inhibitor, is now under evaluation of for the treatment of CRPC and metastatic prostate cancer [53]. 

Retinoblastoma (RB) is a tumor suppressor protein that is frequently disrupted and has been considered as a critical negative regulator of tumor development by preventing cell cycle deregulation [54]. Recently, Sharma et al. presented data revealing the contribution of RB in prostate cancer progression to CRPC. They insisted that (1) RB loss of function was overrepresented in CRPC and metastatic prostate cancer; (2) *RB1* gene loss was frequently observed in CRPC; (3) RB depletion was sufficient to induce castration-resistant tumor growth through AR deregulation; (4) RB depletion resulted in AR mRNA deregulation and protein accumulation through stringent E2F1-mediated regulation; (5) perturbation of the RB/E2F/AR axis was frequently observed in CRPC, resulting in AR upregulation [55].

In human prostate cancer, genomic alterations of ETS-related genes, principally *ERG*, often occur as fusions between an androgen receptor-regulated gene promoter of *TMPRSS2* and ETS transcription factors and are detected in about 50% of tumors [56, 57]. This high frequency of ETS genetic rearrangements led us to investigate contribution of the aberrant ETS expression in prostate tumorigenesis. These prostate cancer-specific gene rearrangements may be explained by the fact that androgen treatment in AR-positive prostate cancer cell lines induced proximity between *TMPRSS2* and *ERG* [58]. According to ERG transgenic mouse models, *TMPRSS2-ERG *alone is insufficient to induce prostate carcinogenesis [59, 60]. However, when they crossed this fusion transgene with *Pten*-deficient mice, they obtained offspring that developed PIN and sometimes invasive adenocarcinoma [59, 60]. The majority of ERG-positive prostate cancer samples had reduced or absent PTEN expression, suggesting that *ERG *rearrangements and loss of *PTEN* were concurrent genetic events [12, 59, 60]. Collectively, two frequent critical events in human prostate cancer cooperate to promote tumor development and progression in prostate cancer. Recently, Yu et al. explored the genomic binding sites of ERG and AR [61]. They found that there is an extraordinary degree of overlap between these two crucial transcription factors binding sites in prostate cancer. Pursing this line of investigation, they found that ERG suppresses AR function through a decrease in AR levels as well as inhibiting AR transcriptional activity. Moreover, they suggested that ERG regulates polycomb group protein EZH2-mediated epigenetic silencing. Thus, ERG may contribute to disruption of androgen-mediated prostatic differentiation and induction of EZH2-mediated cellular dedifferentiation. Additionally, by inhibiting AR signaling, TMPRSS2-ERG may exert a selective pressure for the development of CRPC. Kunderfranco et al. revealed that ERG represses NKx3.1, a prostate-specific homeobox protein involved in prostate development and differentiation, through induction of EZH2 expression [62]. Although this supports work by Yu and colleagues, further characterization of ERG and AR signaling is needed.

## 8. Conclusion

This paper highlights recent advances in understanding the contribution of signal transduction pathways in the progression to CRPC. Although various new agents targeting AR and testosterone synthetic pathways are under evaluation in clinical trials, more effective strategies will be necessary for preventing the transition to lethal states of prostate cancer, and this will require deeper understanding of underlying mechanisms of CRPC.
